# Modulatory role of phospholipase D in the activation of signal transducer and activator of transcription (STAT)-3 by thyroid oncogenic kinase RET/PTC

**DOI:** 10.1186/1471-2407-8-144

**Published:** 2008-05-23

**Authors:** Young-Rae Kim, Hee Sun Byun, Minho Won, Kyeong Ah Park, Jin Man Kim, Byung Lyul Choi, Hyunji Lee, Jang Hee Hong, Jongsun Park, Jeong Ho Seok, Dong Wook Kim, Minho Shong, Seung-Kiel Park, Gang Min Hur

**Affiliations:** 1 Department of Pharmacology, College of Medicine, Chungnam National University, Daejeon, 301-131, South Korea; 2 Department of Pathology, College of Medicine, Chungnam National University, Daejeon, South Korea; 3 Department of Internal Medicine, College of Medicine, Chungnam National University, Daejeon, South Korea; 4 Department of Biochemistry, College of Medicine, Chungnam National University, Daejeon, South Korea; 5 Infection Signaling Network Research Center, Research Institute for Medical Scineces, College of Medicine, Chungnam National University, Daejeon, South Korea

## Abstract

**Background:**

RET/PTC (rearranged in transformation/papillary thyroid carcinomas) gene rearrangements are the most frequent genetic alterations identified in papillary thyroid carcinoma. Although it has been established that RET/PTC kinase plays a crucial role in intracellular signaling pathways that regulate cellular transformation, growth, and proliferation in thyroid epithelial cells, the upstream signaling that leads to the activation of RET/PTC is largely unknown. Based on the observation of high levels of PLD expression in human papillary thyroid cancer tissues, we investigated whether PLD plays a role in the regulating the RET/PTC-induced STAT3 activation.

**Methods:**

Cancer tissue samples were obtained from papillary thyroid cancer patients (n = 6). The expression level of PLD was examined using immunohistochemistry and western blotting. Direct interaction between RET/PTC and PLD was analyzed by co-immunoprecipitation assay. PLD activity was assessed by measuring the formation of [^3^H]phosphatidylbutanol, the product of PLD-mediated transphosphatidylation, in the presence of *n*-butanol. The transcriptional activity of STAT3 was assessed by m67 luciferase reporter assay.

**Results:**

In human papillary thyroid cancer, the expression levels of PLD2 protein were higher than those in the corresponding paired normal tissues. PLD and RET/PTC could be co-immunoprecipitated from cells where each protein was over-expressed. In addition, the activation of PLD by pervanadate triggered phosphorylation of tyrosine 705 residue on STAT-3, and its phosphorylation was dramatically higher in TPC-1 cells (from papillary carcinoma) that have an endogenous RET/PTC1 than in ARO cells (from anaplastic carcinoma) without alteration of total STAT-3 expression. Moreover, the RET/PTC-mediated transcriptional activation of STAT-3 was synergistically increased by over-expression of PLD, whereas the PLD activity as a lipid hydrolyzing enzyme was not affected by RET/PTC.

**Conclusion:**

These findings led us to suggest that the PLD synergistically functions to activate the STAT3 signaling by interacting directly with the thyroid oncogenic kinase RET/PTC.

## Background

Papillary thyroid carcinoma (PTC) is frequently associated with RET (rearranged in transformation) gene rearrangement that results in fusing the RET tyrosine kinase domain to the N-terminal region of heterologous genes [1]. Among human tumors, RET rearrangements are restricted to the thyroid gland, where they are considered specific for papillary carcinoma [2,3]. To date, 12 different fusion partner genes are reported to form at least 17 different RET hybrid oncogenes. The most prevalent variants of these chimeric oncogenes are RET/PTC1 (60 to 70%) and RET/PTC3 (20 to 30%) [1,3-5]. The rearrangements result in ligand-independent activation of RET tyrosine kinase which is considered crucial for activating the cellular signaling pathways involved in transformation of thyroid cells. Indeed, RET/PTC transforms thyroid follicular cells *in vitro *[6], and the specific overexpression of RET/PTC1 or RET/PTC3 in the thyroid of transgenic mice leads to the development of tumors that resemble PTC [7-9].

In recent years, extensive research on the RET/PTC signaling pathway has greatly advanced our understanding of the mechanisms of thyroid tumorigenesis. It has been shown that the transforming potential of RET/PTC is related to the activation of multiple signaling cascade and tyrosine kinase [10]. As the chimeric forms of RET tyrosine kinase are translated into fusion proteins, the protein-protein interaction motifs of amino terminal region allow dimerizing interfaces, which then lead to RET autophosphorylation and constitutive kinase activity. Consequently, when phosphorylated, it acts as a binding site for several adaptors and signaling molecules, including Src homology 2 domain containing (Shc), growth factor receptor binding protein2 (Grb2), insulin receptor substrate 1/2 and phospholipase Cγ [1]. This triggers a cytoplasmic signaling that leads to the activation of Ras/ERK and phosphatidylinositol 3-kinase (PI-3K) signaling pathways [11]. In a recent experiment, the signal transducer and activator of transcription-3 (STAT-3) has been identified as a direct substrate for RET/PTC tyrosine kinase in thyroid cells. In fact, it has been shown that RET/PTC associates with STAT-3 and activates it through the specific phosphorylation of the tyrosine 705 residue, and thus turns on the cyclin D1, vascular endothelial growth factor (VEGF) and intracellular adhesion molecule 1 [12]. These findings suggest that STAT3 activation by the RET/PTC tyrosine kinase is one of the critical signaling pathways for cellular transformation during the condition of thyroid tumorigenesis.

Phospholipase D (PLD) family is a signal transduction-activated enzyme, which is responsible for many cellular signals during a variety of cellular processes, including membrane trafficking, cytoskeletal reorganization, cell proliferation, differentiation, survival and apoptosis [13]. Two isoforms of PLD, PLD1 and PLD2, have been studied in mammalian cells [14,15], and they differ in the mechanism of activation and subcellular localization [14,16]. PLD1 has a low basal activity and is found throughout the cells, but particularly in perinuclear, Golgi and heavy membrane fractions [17,18]. In contrast, PLD2 has a high basal activity and is localized mainly on the plasma membrane [18]. Although PLD1 has likewise been implicated in retrograde vesicle movement in the Golgi and receptor endocytosis [16,19,20], both PLD1 and PLD2 have also reported to provide a survival signal that overcomes cell cycle arrest and apoptotic cell death [21]. Many studies have shown that PLD activity is enhanced in response to platelet-derived growth factor (PDGF) [22], epidermal growth factor (EGF) [23], fibroblast growth factor (FGF) [24], growth hormone and insulin-like growth factor 1 [25]. PLD activity is also elevated in the cells transformed by a variety of transforming oncogenes and some human cancers [21,26]. More importantly, it has been reported that PLD not only suppresses apoptosis in the cells with elevated tyrosine kinase activity but also cooperates with tyrosine kinases to contribute to progressing to a malignant phenotypes [27,28]. This suggests that the elevated PLD activity may have a critical role for cell proliferation, survival signaling, and tumor progression in cancers where elevated tyrosine kinase expression is common. Indeed, the elevated expression or activity of PLD has been reported in breast cancer tissues and cell lines where frequently there is the elevated expression of tyrosine kinases such as the EGF receptor, c-Src and Her2/Neu [29]. Consequent with this idea, we hypothesize that PLD synergistically functions to activate STAT3 signaling by directly interacting with thyroid oncogenic kinase RET/PTC in the papillary thyroid cancer cells.

## Methods

### Tissue samples and preparation of homogenates

Tissue samples were obtained from sequential patients during surgical operations for papillary thyroid cancers at Chungnam National University Hospital, Daejeon, Korea. The study was approved by the hospital institutional review board (approval number 07-04) according to the Declaration of Helsinki, and the written informed consent was obtained from each patient by research team prior to surgery. After washing with cold saline, normal and cancerous tissues were immediately frozen in liquid nitrogen and then stored at -70°C until analysis. Frozen tissues were thawed and homogenized in 100 mM Tris-HCl (pH 8.0) and 1% Triton X-100. Tissue homogenates were centrifuged at 1,500 × g for 15 min to remove debris. Following clarification, the homogenates were centrifuged at 100,000 × g for 1 h at 4°C. Protein concentration of homogenates was determined for each sample using the Bradford method (Bio-Rad, Hercules, CA)

### Cell culture and transfection

HEK293 cells, NIH 3T3 cells, human papillary thyroid cancer cells (TPC1), which bears the RET/PTC1 rearrangement, and human anaplastic thyroid cancer cells (ARO) were cultured in Dulbecco's modified Eagle's medium supplemented with 10% fetal bovine serum, 2 mM glutamine, 100 U/ml penicillin, and 100 μg/ml streptomycin at 37°C with 5% CO_2_. The cells were transiently transfected with the flag-PLD and RET/PTC expression plasmids using lipofectAMINE PLUS (Invitrogen, San Diego, CA) following the manufacturer's instructions. Briefly, the plasmids were mixed with the Plus reagent and then incubated with lipofectAMINE. The lipofectAMINE Plus DNA complex was added to the cells and further incubated for 3 h at 37°C. The control cells received the lipofectAMINE PLUS alone. After incubation, the medium was removed and replaced with fresh medium and cells were maintained for additional 24 h.

### PLD activity assay

PLD activity was assayed by measuring the formation of phosphatidylbutanol (PtdBut), the product of PLD-mediated transphosphatidylation, in the presence of 0.3% *n*-butanol. TPC1 and ARO cells were loaded in complete medium containing 1 μCi/ml of [^3^H]myristic acid for 3 h at 37°C. Cells were then washed twice with phosphate-buffered saline (PBS) and preequilibrated in serum-free DMEM for 1 h and treated for the indicated concentrations with pervanadate. For the final 10 min of preincubation, 0.3% *n-*butanol was added. Cells were then collected and the lipids were extracted according to the method of Lee [30]. Briefly, PtdBut was separated by thin layer chromatography (TLC) on silica gel 60A plates using a solvent complex of ethylacetate/isooctane/acetic acid/water (110/50/20/11, v/v). The region corresponding to PtdBut was visualized by iodine vapor staining, scraped, and counted by a liquid scintillation counter (Beckman LS 6500). Radioactivity incorporated into total lipids was used to normalize the results. The produced [^3^H]PtdBut by PLD activity was expressed as a ratio of total lipid radioactivity ([^3^H]PtdBut/total lipid).

### Western blot analysis

After treating with different reagents as described in the Figure legends, cells were collected and lysed in M2 buffer (20 mM Tris, pH 7.6, 0.5% NP-40, 250 mM NaCl, 3 mM EDTA, 3 mM EGTA, 2 mM dithiothreitol, 0.5 mM PMSF, 20 mM β-glycerol phosphate, 1 mM sodium vanadate, and 1 μg/ml leupeptin). Then the lysates were denatured by boiling for 5 min in 1 × SDS-PAGE sample buffer, resolved on sodium dodecyl sulfate-polyacrylamide gel and blotted onto polyvinylidene difluoride membrane. After blocking with 5% skim milk in PBS containing 0.05% tween-20, the membrane was probed with the relevant antibodies and visualized by enhanced chemiluminescence (Amersham, Arlington Heights, IL).

### RT-PCR

Expression of RET/PTC in papillary thyroid cancer tissues were analyzed by semiquantitative RT-PCR analysis. After the total RNA was prepared using a TRIZOL reagent (Life Technologies, Inc.), the oligo(dT)-primed cDNA was synthesized using a RT-PCR kit (Stratagene, La Jolla CA). PCR amplification was carried out using the primer pair specific to human RET/PTC1 (5'-GTCGGGGGGCATTGTCATCT-3' and 5'-AAGTTCTTCCGAGGGAATTC-3') and human GAPDH (5'-GACCCCTTCATTGA CCTC-3' and 5'-GCCATCCACAGTCTTCTG-3')

### Immunoprecipitation assay

For immunoprecipitation assay, the cells were collected in lysis buffer (50 mM HEPES at pH 7.6, 150 mM NaCl, 0.1% NP-40, 5 mM EDTA, 0.5 mM phenylmethyl sulfonyl fluoride, 1 μg/ml leupeptin, 1 μg/ml aprotinin and 1 μg/ml pepstatin) after treatments as described in the Figure legends. The resulting cell lysates were spun at 15,000 × g for 10 min at 4°C, and protein the concentration of the supernatants were measured by Bradford method with a bovine serum albumin as a standard. An equal amount of cell lysates was precipitated with the relevant antibody (1 μg) against flag or RET and protein G-sepharose beads by incubation at 4°C for 4 h to overnight. All the immunoprecipitates were washed four times with lysis buffer, boiled in SDS-PAGE sample buffer and resolved on 10% polyacrylamide gel.

### Immunohistochemistry

Expression of PLD was analyzed by immunohistochemistry (IHC) on paraffin-embedded tissue sections from six thyroid cancer tissues. Three μm thick sections from the paraffin blocks were used for IHC with rabbit EnVision-HRP detection system (Dako, Crpinteria, CA, USA). The anti-PLD antibody (Upstate, Lake Placid, NY) was used for IHC. After deparaffinization and antigen retrieval by a pressure cooker in 10 mM sodium citrate buffer (pH 6.0) at full power for 4 min, tissue sections were treated with 3% hydrogen peroxide for 10 min. The primary antibody was diluted (1:50) with background reducing diluent (Dako) and incubated for 30 min. The slides were then incubated with the En Vision reagent for 30 min, and then sequentially incubated with DAB chromogen for 5 min, counterstained with Meyer's hematoxylin and mounted. Careful rinses with several changes of TBS-0.3% tween buffer were performed at each step. Negative control was used using mouse IgG1 isotype control to exclude the primary antibody. Cytoplasmic staining was considered as positive cells.

### Luciferase assay

Firefly and *Renilla *luciferase activities were measured with the Dual-Luciferase assay system. After transfection with *m*67-Luc, pRL-SV40 that is encoding *Renilla *luciferase, STAT3, RET/PTC and PLD construct, the cells were lysed and the luciferase assay was performed using a luciferase assay kit (Promega Corp., Madison. WI). The data were presented as fold inductions of the ratio of firefly per *Renilla *luciferase activities of each sample.

### Statistical analysis

Data are expressed as the mean ± SE from at least three separate experiments performed in triplicate. The differences between groups were analyzed using a Student' s t-test, and P values < 0.05 were considered statistically significant. Statistical analyses were carried out using Statistical Package for Social Science software program (version 11.0; SPSS Inc. IL.).

## Results and Discussion

### PLD2 level is increased in human papillary thyroid cancer tissues

**Figure 1:**
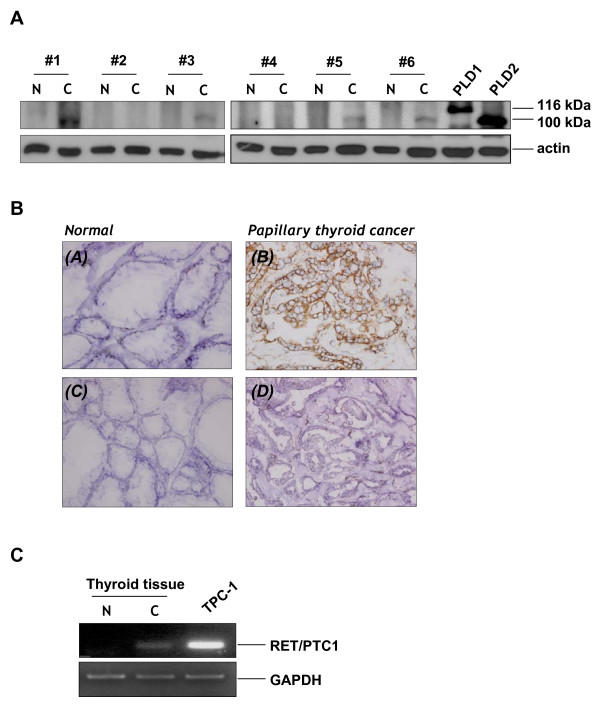
**Overexpression of PLD2 in papillary thyroid tumor tissue**. (A) Normal and cancer tissue extracts from 6 patients who underwent surgery for malignant papillary thyroid cancer were prepared as described in Methods. The tissue extracts and transfectant controls of PLD1/PLD2 were applied to SDS-PAGE for Western blotting with antibodies directed against PLD and β-actin. (B) Immunohistochemistry of PLD in normal and cancerous tissue. PLD2 was highly expressed in papillary thyroid cancer tissue (*B*) compared to faintly stained normal thyroid tissue (*A*). (*C, D*) Negative isotype control with preimmune mouse IgG1. Original magnification, 100×. (C) Expression of RET/PTC-1 in malignant papillary thyroid cancer tissue. After the normal and cancer tissues were lysed and the total RNA was isolated, RT-PCR was performed with the primers specific to the human RET/PTC1 and GAPDH. After the PCR amplification, the products were separated by agarose gel electrophoresis and visualized using ethidium bromide staining. The results are representative of three independent experiments.

Since PLD contributes to the progression to a malignant phenotype in the cells with elevated tyrosine kinase activity [27], the level of PLD expression may have a close relationship with the development of papillary thyroid cancers. Therefore, the expression levels of PLD were compared by Western blotting in normal and cancerous tissues from 6 patients who underwent surgery for papillary thyroid cancer. As shown in Figure 1A, we detected the enhanced expression of PLD2 in 4 out of the 6 papillary thyroid cancer tissues, though PLD1 which is predicted to be 116 kDa, was not detected in the same blot. PLD2 was low or nondetectable in normal thyroid tissues, suggesting that the enhanced PLD expression, especially in PLD2, may play a role in controlling the papillary thyroid cancer development. To confirm this observation, we performed immunohistochemistry on the same tissues. As expected, the papillary thyroid cancer tissues expressed PLD2 at a dramatically higher level than the normal tissue (Fig. 1B). To ensure that RET/PTC is indeed express in papillary thyroid cancer tissues, we confirmed the expression of RET/PTC-1 by semiquantitative RT-PCR in the cancerous tissues but not in normal thyroid tissues (Fig. 1C). The mRNA expression of papillary (TPC-1) thyroid cancer cell lines, which harbor RET/PTC1 was served as a control.

### PLDs physically interact with RET/PTC1 and synergistically enhance in STAT3 phosphorylation induced by RET/PTC tyrosine kinase

**Figure 2:**
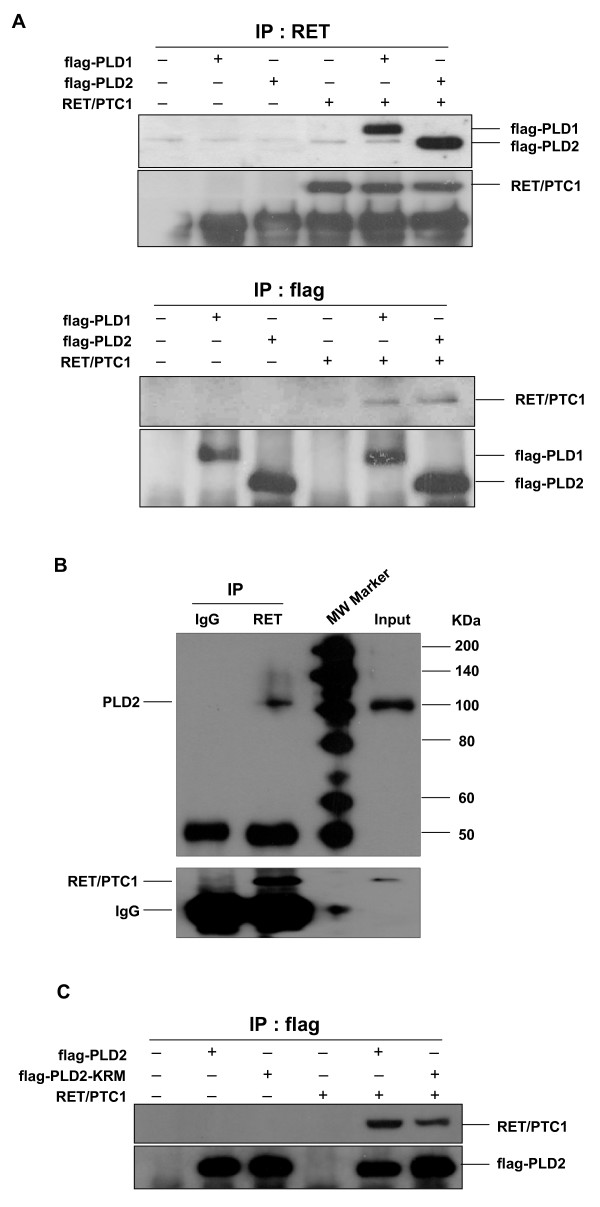
**Physical association between PLD and RET/PTC1**. (A) HEK293 cells were transfected with flag-tagged-PLD1 or -PLD2 and RET/PTC1 expression plasmids. After 24 h transfection, whole cell lysates were immunoprecipitated with anti-RET (upper panel) or anti-flag (lower panel) antibodies, and then immunoprecipitants were analyzed by Western blotting with anti-flag or anti-RET antibodies. The efficiency of immune-precipitant was measured with anti-RET and anti-flag antibodies (each bottom panel). (B) TPC-1 cells were transfected with RET/PTC1 expression plasmid, and whole cell lysate was immnunoprecipitated with antibodies directed against RET and normal IgG. Immunoprecipitates were analyzed by Western blotting with anti-PLD antibody (upper panel). The efficiency of immune-precipitant was measured with anti-RET antibody (bottom panel). One percent of cell extracts from sample was used as a control of protein input. (C) HEK293 cells were transfected with various combinations of plasmids encoding an empty vector, RET/PTC1 plus either flag-tagged wild-type (wt)- or lipase inactive mutant (KRM)- PLD2. Cell lysates were immunoprecipitated with anti-flag antibody, and then immunoprecipitants were analyzed by Western blotting with anti-RET antibody (upper panel). The efficiency of immune-precipitant was measured with anti-flag antibody (bottom panel).

Results of recent studies have indicated that the elevated expression of PLD cooperates with non-receptor tyrosine kinase, such as c-Src to induce the cellular proliferation through direct interaction [28]. Since RET/PTC tyrosine kinase is linked to the specific signaling pathway in papillary thyroid cancer, we examined whether PLD isotypes could associate with the RET/PTC. Cells were cotransfected with PLD1 or PLD2 plus RET/PTC1 in HEK293 cells, and the cell lysates were immunoprecipitated with antibodies against RET or Flag. Coimmunoprecipitation assays indicated that both PLD1 and PLD2 associated with RET/PTC1 to a similar extent when the cell lysates were immunoprecipitated with antibodies against RET or Flag (Fig. 2A). Importantly, endogenous PLD2 was found to interact consistently with RET/PTC1 in TPC-1 cells, where RET/PTC1 protein was overexpressed (Fig. 2B). Another oncogenic form, RET/PTC3, also interacted with PLD1 or PLD2 (data not shown), suggesting that the interaction of PLD and RET/PTC takes place presumably through catalytic domain of RET. In previous studies, it has been shown that the enzymatic activity of PLD as a lipid hydrolytic enzyme is not be strictly required for PLD function to amplify the mitogenic signal [28,31]. Therefore, we next investigated whether the lipase activity of PLD is required for interaction with RET/PTC-1. The lipase-inactive mutant of PLD2, PLD2-K758R (KRM) also bound to RET/PTC1 as like the wild-type PLD2 (Fig. 2C), indicating that the lipase activity of PLD2 is not required for this interaction.

**Figure 3:**
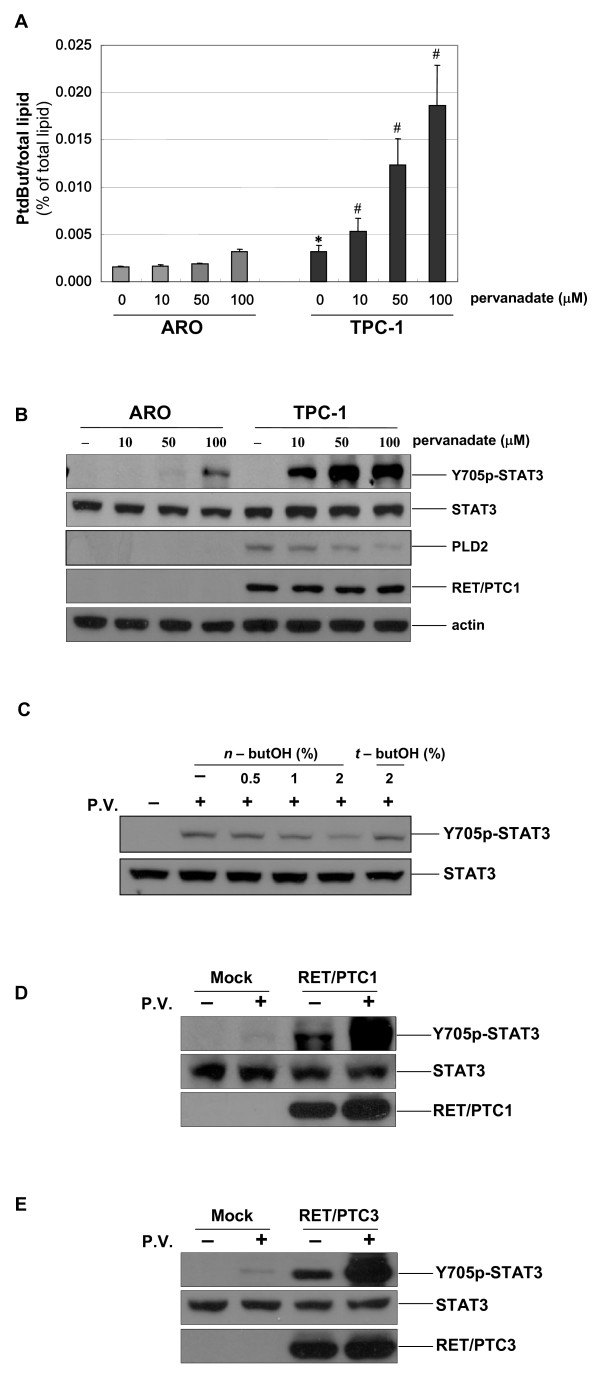
**PLD synergistically enhanced in STAT3 phosphorylation induced by RET/PTC tyrosine kinase**. (A) ARO and TPC-1 cells were prelabeled with [^3^H]-myristic acid for 3 h and then treated with the various concentrations of pervanadate for 30 min as indicated, 10 min before the end of the treatment, cells were incubated with 0.3% *n*-butanol. Radioactivity incorporated into PtdBut was measured as described in Methods. Each bar shows mean ± SE of at least of three independent experiments. * *P *< 0.05, when compared with none-treated ARO cells. # *P *< 0.05, when compared with pervanadate-treated ARO cells. (B) ARO and TPC-1 cells were treated with the various concentrations of pervanadate for 30 min as indicated. Whole cell lysates were analyzed by SDS-PAGE and Western blotting with antibodies against phosphorylated STAT3 (Y705), RET and PLD2. To confirm equal amounts of protein were loaded, the blots were reproved using an antibody against total STAT3 and β-actin. (C) TPC-1 cells were pretreated with *n*-butanol for indicated concentrations or 2% *t*-butanol and then treated with 10 μM pervanadate for 30 min. Cell extracts were analyzed by SDS-PAGE and Western blotting with antibodies against phosphoY705-STAT3 and STAT3. (D, E) ARO cells were transiently transfected with mock vector and RET/PTC1 (D) or RET/PTC3 (E) expression plasmids. At 24 h transfection, the cells were untreated or treated with 50 μM pervanadate for 30 min, and then total cell lysates were prepared and blotted with anti-phosphoY705-STAT3, anti-STAT3 and anti-RET antibodies. The figure is representative of at least three separate experiments.

It has been well established that TPC-1 cell lines has cytogeneticallay genomic changes characterized by translocation involving chromosome 1, 10 and 21 (RET/PTC rearrangement) and therefore this cell atypically harbors RET/PTC1 among the thyroid epithelial cancer cells [32]. To address the biological relevance of high PLD expression and RET/PTC in papillary thyroid cancers, we compared the activity of PLD and the extent of pervanadate-induced STAT3 phosphorylation in human anaplastic (ARO) and papillary (TPC-1) thyroid cancer cell lines. The basal activity and pervanadate-induced activation of PLD were significantly higher in TPC-1 cells than in ARO cells (Fig. 3A). Furthermore, we also observed the enhanced expression of PLD2 in TPC-1 cells compared to that in ARO cells (Fig. 3B, third panel). This is consistent with the results shown earlier that PLD2 level is increased in papillary thyroid cancer tissue (Figs. 1A and 1B). Previously, it has been indicated that STAT3 is a direct substrate of RET/PTC tyrosine kinase and that the phosphorylation of tyrosine (Y) 705 in STAT3 by RET/PTC is critical for the RET/PTC-mediated transformation process [12]. More importantly, under these conditions, the immunoblotting with the antibody specific to phosphorylated Y705 of STAT3 showed that the extent of pervanadate-induced STAT3 phosphorylation was also much higher in TPC-1 cells, without alteration of total STAT-3 expression (Fig. 3B, first and second panel). To ensure that the expression of RET/PTC1 is indeed defective in ARO cells, the cell lysates obtained from ARO cell and TPC1 cells were immunoblotted with antibody against RET/PTC1 and actin (Fig. 3B, fourth and bottom panel). Furthermore, when TPC-1 cells were pretreated with the PLD-specific inhibitor *n*-butanol, pervanadate-induced STAT3 phosphorylation was decreased, in a dose-dependent manner (Fig. 3C), indicating that pervanadate-induced STAT3 phosphorylation is indeed mediated by PLD in TPC-1 cells.

To strengthen our findings, ARO cells were transfected with pCMV-track-RET/PTC or control pCMV-track, and subsequently treated with pervanadate to induce PLD activation. We detected the Y705 phosphorylation of STAT3 after transfection with RET/PTC1 (Fig. 3D) and RET/PTC3 (Fig. 3E), which is consistent with a previous report [12]. As a control, the cells transfected with mock vector did not show Y705 phosphorylation of STAT3. Furthermore, we also found that the pervanadate treatment resulted in a dramatically higher level of Y705 phosphorylation of STAT3 under overexpression conditions of RET/PTC1 or RET/PTC3 in ARO cells (Fig. 3D and 3E). These data provide additional evidence that the activation of PLD enhanced by pervanadate contributes to RET/PTC-mediated STAT3 phosphorylation in thyroid epithelial cells.

### RET/PTC enhances tyrosine phosphorylation of PLD2, but it is not necessary for functional PLD activity

**Figure 4:**
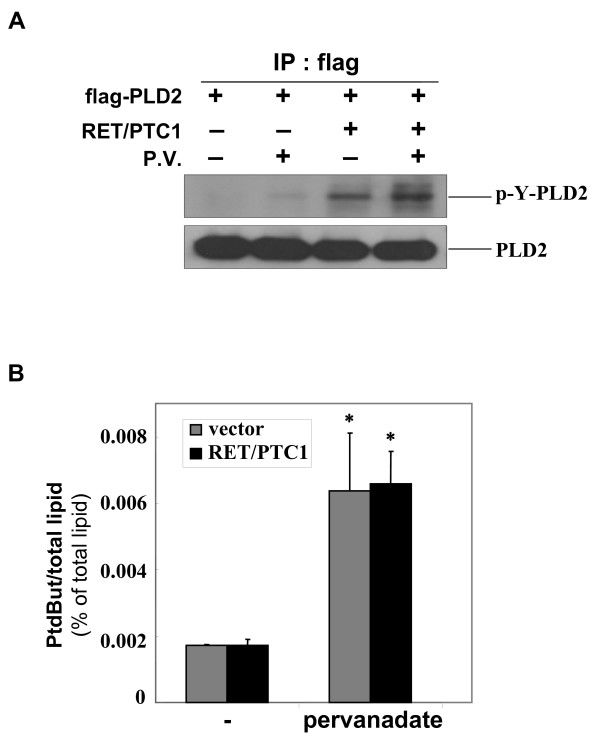
**RET/PTC enhances tyrosine phosphorylation of PLD2, but it does not affect the enzyme activity of PLD**. (A) HEK293 cells were transiently transfected with mock vector, flag-PLD2, RET/PTC1 expression plasmids, treated with pervanadate (50 μM) for 30 min, and subjected to immunoprecipitation with anti-flag antibody. Immunoprecipitate was analyzed by Western blotting with anti-phosphotyrosine specific antibody (4G10), and the efficiency of immunoprecipitation was measured with anti-PLD2 antibody. (B) NIH3T3 cells were transiently transfected with mock vector and RET/PTC1 expression plasmids, treated with pervanadate (50 μM) for 30 min. PLD activity was measured as described in the legend of Fig. 3A. Radioactivity of PtdBut was expressed in percent of radioactivity of total phospholipids. * *P *< 0.05, when compared with empty vector transfected group. Each bar shows mean ± SE of at least of three independent experiments.

The tyrosine kinase activity of RET/PTC is a crucial for its oncogenic potential and for activation of the intracellular signaling pathways involved in transformation [33]. Since we found the physical interaction between PLD and RET/PTC (Fig. 2), we examined whether RET/PTC tyrosine kinase is capable of phosphorylating PLD. Immunoprecipitation assays were performed with Flag-tagged PLD2 in HEK293 cells cotransfected with RET/PTC1 and/or Flag-PLD2, and an immunoblot analysis was conducted with the antiphosphotyrosine antibody, 4G10. As shown in the upper panel of Figure 4A, we found the tyrosine phosphorylation of PLD2 in the cells cotransfected with RET/PTC1 whereas the minimal basal tyrosine phosphorylated protein was detected in the cells transfected with mock vector. Furthermore, treatment with pervanadate, a PLD activator, also induced the tyrosine phosphorylation of PLD2, and the prevanadate-induced tyrosine phosphorylation was enhanced under the RET/PTC1 overexpression condition, suggesting that PLD2 is tyrosine phosphoylated by the expression of RET/PTC tyrosine kinase. To ensure that the same amount of PLD2 was precipitated in each sample, PLD2 levels in the immunoprecipitate were measured (Fig. 4A, lower panel).

Next, we investigated whether the tyrosine phosphorylation of PLD by RET/PTC has any effect on its phopholipase activity. RET/PTC1 was ectopically expressed in NIH3T3 cells, and *in vivo *PLD activity was measured following the pervanadate treatment. Unexpectedly, the pervanadate-induced PLD activity in the cells transfected with RET/PTC1 was similar to that in the cells transfected with mock vector (Fig. 4B). Transient transfection of RET/PTC3 also did not affect the PLD activity (data not shown). These results suggest that the tyrosine phosphorylation of PLD by RET/PTC does not increase the PLD phospholipase activity. However, the biological significance of tyrosine phosphorylation of PLD enhanced by RET/PTC still remains to be determined, because the PLD enzyme activity has not been correlated with its phosphorylation level in this study. Recently, it has been reported that tyrosine phosphorylation on PLD contributes to the cellular proliferation through mitogenic signaling pathways [28,34]. Therefore one can speculate that, in case of formation of binding complex between PLD2 and RET/PTC, tyrosine phosphorylation of PLD2 by RET/PTC might be involved in the potentiation of RET/PTC-mediated STAT3 to amplify the mitogenic signal in papillary thyroid tumor.

### PLD enhances RET/PTC-dependent STAT3 transcriptional activity

**Figure 5:**
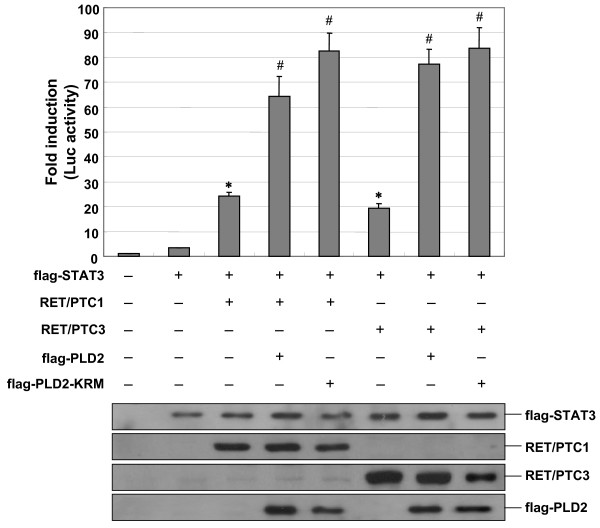
**PLD synergistically enhances RET/PTC-mediated transcriptional activation of STAT3**. HEK293 cells were transfected with various combinations of plasmids encoding an empty vector, STAT3, RET/PTC1, RET/PTC3, flag-tagged wild-type (wt)- or lipase inactive mutant (KRM)- PLD2 and m67 luciferase reporter plasmid. After 24 h transfection, the cells were lysed and the luciferase activities were measured as described in Methods, and the activity of each sample was normalized according to *Renilla *luciferase activity (upper panel). Results show mean ± SE of at least of three independent experiments. * *P *< 0.05, when compared with STAT3 alone-expressed group. # *P *< 0.05, when compared with STAT3 and RET/PTC1- or RET/PTC3-expressed group. To confirm the expression level of each plasmid in transfected cells, the total cell lysates were analyzed by SDS-PAGE and Western blotting with antibodies against flag and RET (lower panel).

In our experiments, we demonstrated that PLD synergistically increases the STAT3 phosphorylation induced by RET/PTC tyrosine kinase, as measured by enhance Y705 phosphorylation of STAT3. Y705 phosphorylation of STAT3 is known to be important for the dimerization, translocation and transcriptional activation of STAT3 [35]. To determine whether the RET/PTC-mediated STAT3 phosphorylation enhanced by PLD could activate the STAT3 transcriptional activities, we co-transfected the cells with plasmids expressing RET/PTC and m67-luciferase reporter construct containing multimerized STAT3-specific binding sites in HEK293 cells. As expected, the transcriptional activity of STAT3 increased up to 20-fold in the cells transfected with RET/PTC1 and RET/PTC3 (Fig. 5). These observations were also confirmed in ARO cells, even though the extent of fold activation was lesser than in HEK293 cells because of a lower transfection efficiency (data not shown). This indicates that the activation of STAT3 can be monitored in transiently transfected cells with the m67-luciferase reporter plasmid. Next, the ability of PLD to regulate the activation of STAT3 was examined in transiently transfected HEK293 cells expressing the m67-luciferase reporter in the absence or presence of plasmids expressing Flag-tagged PLD2. Consistent with the results described above (Fig. 3B, 3D and 3E), the expression of PLD2 synergistically enhanced RET/PTC1- or RET/PTC3-depedent activation of STAT3. Furthermore, the expression of the lipase inactive mutant PLD2 (KRM) enhanced STAT3 activity to a similar extent than did wild-type PLD2 (Fig. 5), which suggest that the lipase activity of PLD2 is unlikely to be involved in RET/PTC-mediated STAT3 activation. These findings led us to conclude that the PLD enhance RET/PTC-mediated STAT3 phosphorylation resulting in the transcriptional activation of STAT3.

In the present study, we hypothesized that the elevated expression of PLD might have a role to trigger a cytoplasmic signaling initiated by a protein tyrosine kinase RET/PTC in papillary thyroid cancer. However, we did not observe the direct evidence of the correlation between elevated expression of PLD and thyroid epithelial growth/differentiation. This could be explained as like that only PLD may be not enough for thyroid tumorigenesis but necessary. Other factors, such as environmental condition, genetic alterations, or hormonal effects may cooperatively responsible for cellular proliferation or differentiation in thyroid epithelial cancer cells.

## Conclusion

In summary, this study shows that the elevated expression of PLD in papillary thyroid cancers plays a synergistic role in the transcriptional activation of STAT3 by interacting with RET/PTC. Thus, our findings offer a significant advance in understanding the cross talk between PLD and thyroid oncogenic kinase RET/PTC during the papillary thyroid tumorigenesis.

## Abbreviations

PLD: phospholipase D; RET/PTC: rearranged in transformation/papillary thyroid carcinomas; STAT: signal transducer and activator of transcription; PtdBut: phosphatidylbutanol.

## Competing interests

The authors declare that they have no competing interests.

## Authors' contributions

Y–RK participated in the design of the study, carried out bench experiments and drafted the manuscript. HSB, MW, KAP, BLC, HL helped carrying out bench experiments related to this study. JMK carried out immunohistochemical examinations. JHH, JP and JHS provided intellectual input in drafting of the manuscript. DWK and MS provided material for this study and helped drafting the manuscript by providing critical intellectual input. SKP participated in the design of the study, provided material for this study and helped drafting the manuscript. GMH designed this study, interpreted the results, helped drafting the manuscript, and finalized the manuscript after input from the other authors.

## Pre-publication history

The pre-publication history for this paper can be accessed here:


